# A New Glycosuric Reagent

**Published:** 1878-06

**Authors:** 


A New Glycosuric Reagent.—Every one knows the uncertainty of Trommer's test and the bother of Fehling's solution. The following will be found a convenient substitute:

Take of

| | |
|---|---|
| Sulphate of copper (ch. pure) . . . | 1 part |
| Crystalized tartrate of soda and potassa . . | 5 parts. |
| Sodic dydrate (ch. pure) . . . . . | 2 " |

Mix thoroughly in a mortar; the more labor spent on this, the better the product. The result will be a pasty mass, which can be transferred to a wide-mouthed bottle, and kept till wanted. To use it, take of the mass a piece about the size of a co. cath. pill, put it in a test tube, and add about two fluid drachms of water; boil till the mass is dissolved, and the solution has a uniform pale and rather dirty blue color; then add two or three drops of the suspected urine and boil again for a moment. If sugar be present, the usual reaction will be manifest. Mr. F. A. Reichardt has succeeded in making the mass into pills of proper size, one pill being sufficient for a test. A vial of these will be handy in the office, and less troublesome to manage than the usual tests. Trommer's test, unless performed with more than usual care, I do not consider reliable; Fehling's solution is reliable, but troublesome. The appreciation of these facts led me to the formula here given, which some one will probably still further improve.—*Dr. Piffard in the Medical Record.*

